# A ‘training of trainers’ programme for operational research: increasing capacity remotely

**DOI:** 10.1080/16549716.2023.2297881

**Published:** 2024-01-15

**Authors:** Angela Willemsen, Eskinder Wolka, Yibeltal Assefa, Simon Reid

**Affiliations:** a School of Public Health, The University of Queensland, Herston, Queensland, Australia; b National Primary Health Care, International Institute for Primary Health Care, Addis Ababa, Ethiopia

**Keywords:** Capability building, hybrid training, practical research, skill sharing, operational research, SORT-IT

## Abstract

**Background:**

Operational research (OR) is a process to improve health system capacity by evaluating interventions to improve health delivery and outcomes. The World Health Organization (WHO) Structured Operational Research Training Initiative (SORT-IT) programme promotes how OR contributes to improved health care delivery and health outcomes. A partnership project between the International Institute of Primary Health Care (IPHCE) in Ethiopia and The University of Queensland (UQ) in Australia modified the SORT-IT programme to deliver a hybrid Training of Trainers programme and improve OR capacity.

**Objective:**

This study was performed to develop and evaluate the effectiveness of Train-the Trainers approach in building capability to expand the capacity of the IPHCE to deliver the SORT-IT programme.

**Methods:**

Recruitment of participants and training were aligned with the principles of the SORT-IT programme. Training was face-to-face for the first session with subsequent training sessions delivered via Zoom over a 13-week period. Participants were required to complete all activities in line with SORT-IT deliverables. Slide decks supporting the SORT-IT training videos were developed and adapted to the Ethiopian context.

**Results:**

Participants had diverse experience from programme directors to research officers. All training sessions were recorded and available for participants to watch and review when required. All participants completed OR protocols to the draft stage. Course evaluation revealed participants found the content and format of the training useful, pertinent, and interesting.

**Conclusion:**

A hybrid model (face-to-face and video platform) for OR training was implemented. Managing contextual challenges such as information technology were managed easily by programme staff. Translating course requirements at a management level proved challenging with data collection for the protocols but provided insight into potential future challenges. This OR Training of Trainers course demonstrated that sharing of skills and knowledge can occur through a hybrid delivery model and contribute to developing capacity.

## Introduction

Operational research (OR) in public health is research that aims to build the evidence-base to enhance the quality, coverage, effectiveness or performance of the health system or disease programme in which research is being conducted [,^1^]. Publishing the results of health interventions allows protocols and policies to be developed and shared with other communities [2,3] and adopted and adapted to further improve health outcomes [2,3]. This process of identifying and documenting programmes contributes to identifying national health objectives [4] as well as contributing to the achievement of sustainable development goals [5].

The Structured Operational Research Training Initiative (SORT-IT) is a capacity building training programme coordinated by the Special Programme for Research and Training in Tropical Diseases (TDR) of the World Health Organization (WHO). This initiative supports the widespread use of OR to contribute to improving health care delivery and outcomes [6,7]. The programme provides an opportunity to highlight programmes that demonstrate improved health outcomes, as well as identifying limitations to greater success. An evaluation of SORT-IT demonstrated significant success in terms of influencing policy at a local and national level in many countries. Initiating and maintaining good stakeholder relationships and involving policy makers throughout the protocol development process were found to have a positive influence on policy development. Barriers to policy development were varied with competing policy influences and even cultural beliefs limiting the inclusion of local findings at a national level [3,8–10].

A twinning project between the International Institute for Primary Health Care in Ethiopia (IPHCE) (https://iphce.org/about/) and The University of Queensland (UQ) in Australia was established to improve the capacity of IPHCE to support improvements in the implementation of primary healthcare in Ethiopia as well as other low- and middle-income countries. A key activity in the project was to upskill IPHCE and its hubs to establish the capacity to deliver OR training. A ‘Training of Trainers’ (ToT) approach was used to accommodate the varied and diverse skills of participants, the local contexts, the desire to scale up training activity and ultimately contribute to strengthening the health system [11]. A ToT approach was purposely used to allow training of a cohort in a compressed time frame, as opposed to a year of experiential learning [12]. This was necessary because the traditional SORT-IT programme relies on the use of a mentorship model to provide participants with hands-on experience, which requires a substantial investment in terms of human resources [13]. The use of video conferencing as an educational medium was rapidly adopted with the ongoing travel limitations imposed by SARS CoV2. The project benefitted from this and believe that video conferencing is a suitable alternative for Training of Trainers [14]. One of the roles of IPHCE is to increase capacity of primary health care (PHC) systems within Ethiopia. Supporting district health managers and health care workers with OR training will help identify, monitor, evaluate and communicate successes and challenges in the implementation of interventions in PHC programmes in Ethiopia. This study was performed to evaluate the effectiveness of this ToT approach in building capability to expand the capacity of the IPHCE to deliver the SORT-IT programme.

## Methods

The TDR SORT-IT OR training and programme [15] model was used as the framework for the OR training (https://tdr.who.int/activities/sort-it-operational-research-and-training). A ‘Training of Trainers’ (ToT) model was used to upskill selected IPHCE and its hubs and prepared them to train district health managers and health care workers in OR.

The IPHCE was founded by the Federal Ministry of Health in 2016 with the aim of improving the PHC system and meeting the Sustainable Development Goals [16]. This will be achieved by serving as a global resource for PHC, training and information sharing, conducting implementation research and contributing to global advocacy [17].

### Recruitment of participants and mentors

Staff from IPHCE and affiliated academic organisations were invited to attend OR ToT by the IPHCE PHC programme lead. A minimum requirement of a Bachelor’s degree was required for participants. All facilitators/trainers and participants were fluent in English as training was delivered in English. The WHO SORT-IT team (https://tdr.who.int/activities/sort-it-operational-research-and-training) were contacted via email to advise them of the proposed OR ToT to be conducted in Ethiopia. The WHO SORT-IT team contacted recent SORT-IT graduates from Ethiopia to connect the training team with graduates interested in acting as SORT-IT mentors. The mentors were invited to all ToT sessions, were given access to the learning management system (BlackBoard®) and included in any email correspondence from the facilitators. The ToT participants established a messenger group using the Telegram Messenger app [18] to allow communication between participants and facilitators.

### Training of trainer training approach

Training for trainers in the three SORT-IT modules (https://tdr.who.int/activities/sort-it-operational-research-and-training) was distributed across seven synchronous sessions delivered using Zoom. Each session included a formal presentation and activities that complemented the module objectives and key facilitation challenges.

The first session hosted by IPHCE included participant introductions (i.e. their background, research and training experience), an icebreaker activity and information on the course structure and requirements. Additional activities were conducted to ensure all participants were able to download and use Zoom. Participants were advised of the course expectations that included self-study of resources and readings as listed in the ToT Facilitators Manual.

The writing of an OR protocol was used as one of the processes for trainers to understand the process that their future students would need to undergo. Trainers and mentors were allocated to four groups where they selected and worked on a suitable OR project with the aim of completing all milestones and publishing the protocol. The OR protocols were developed according to PHC need within geographical regions within Ethiopia. These regions were represented by participants completing the OR ToT programme who were also staff from one of the affiliated universities. Participants had time allocated during all training sessions to discuss and work towards their protocols. Participants understood that completion of the protocols would not be possible due to the expedited time frame of the training (13 weeks) when compared with the SORT-IT programme (10 months). A USB stick containing all training materials (PowerPoint slide decks, ToT Facilitator Manual, Excel Training videos) and references was provided at the completion of the ToT training.

### Training delivery

The ToT training was delivered using a combination of face-to-face and live Zoom video conferences (see Table 1). The training sessions were two-hour duration and scheduled for 09:00 hours (East Africa time (EAT, GMT + 3)/16:00 Australian Eastern Standard Time (AEST, GMT + 10). The first training session was delivered in person by one of the two UQ facilitators in person at the IPHCE, with the other facilitator attending live via Zoom. Subsequent training sessions were all delivered via live Zoom with both facilitators in attendance. Training consisted of formal presentations, small group discussions and practical activities. All training sessions were recorded and made available with their corresponding PowerPoint® slides on BlackBoard®. This system became a repository for other information including the timetable, journal articles and training resources such as ethics forms.

**Table 1.** Summary of training-of-trainers SORT-IT sessions (module 1 – operational research and protocol development, module 2 – data collection and management, module 3 – preparing the report and translation).

Week	Session number	SORT-IT modules	Mode	Duration	Comments
1	1	Introduction and Welcome	Face-to-face	Within session 1	Introduction of trainers, facilitators, icebreaker activity and video conferencing refresher
		Module 1: Day 1	Face-to-face	4 hours	Training conducted in IPHCE with one trainer. One trainer participated via Zoom
2	2	Module 1: Day 2	Online	3 hours	
3	3	Module 1: Day 3	Online	1.5 hours	
4–7	Participants work on ethics submission and protocol development				
8	4	Module 1: Days 4 and 5	Online	2 hours	
9	5	Module 2: Data collection and management	Online	2 hours	
10	6	Module 3: Preparing the report and translation	Online	2hours	
11–12	Participants work on protocol development				
13	7	Module 3	Online	2 hours	

Each training session began with a brief recap of the previous session and an opportunity for participants to ask questions or facilitators to respond to any questions received since the previous session. Sessions concluded with time for questions relating to the training as well as access to course resources and/or technology issues. A face-to-face three hour ‘Good practice guide to learning and teaching’ workshop was facilitated by Learning Designers during a one-week Study tour to Australia. The interactive workshop presented sessions on curriculum design, authentic assessment and active learning. The aim of the workshop was to provide participants with practical experience in engaging students and delivering meaningful learning experience.

### Similarities and differences between the TOT and the SORT-IT or program

The IPHCE course was designed to maintain the SORT-IT model of restricting each cohort to a total of 12 participants and four facilitators/mentors (one per three participants). The structure of the modules was unchanged with milestones, student plenary presentations and links to SORT-IT training videos included in the training package. The duration of each of the three modules was reduced to five days rather than six, six and eight days duration, respectively. Microsoft Excel® was used in place of EpiData for data analysis. The UQ Digital Capability Support developed four Microsoft Excel® training (MP4 format) videos (introduction, charts, processing data and further functions) for use as training material for future OR participants. Participants had the option of using EndNote or Mendeley Reference Management Systems. The IPHCE has an onsite library and librarian for support as required.

### Operational research training of trainer resources

The SORT-IT modules, milestones, criteria and video lectures were used as the foundation for the OR training. The three SORT-IT modules are as follows: 1. Protocol development, 2. Data capture and analysis and 3. Manuscript writing and publication [19]. A ToT and Facilitators Manual was developed based on these modules and included practical information such as adult learning principles [20], as well as ice breaker activities, course timetable, student pre-requisites, course delivery information, evaluation templates and logistical information to implement future courses in Ethiopia. A summary of the information is contained in Table 2.

**Table 2.** Contents of training of trainers and facilitators manual (for operational research training).

Training of Trainers and facilitators Manual chapters	Chapter contents
Introduction, definitions, target audience	
Preparing for training	Planning and organising the workshop Funding considerations Participant requirements
Training basics	Facilitator needs and expectations
Timeline for Operational Research Training (based on SORT-IT model)	Communication Group work Engaging participants Adult learning principles Icebreakers Ground rules Certificates of completion
Module 1 – Operational Research and protocol development	Aim Objectives Pre-requisites Expected duration Expected outcomes Outputs Lectures Workshops Resources
Module 2 – Data collection and management	As for Module 1
Module 3 – Preparing the report and translation	As for Module 1
Appendices	Code of Conduct (for students) Pre-course evaluation Course completion certificate template Timetable for Module 1, 2 and 3 Critical reading and analysis Grey literature

Resources for the participants were developed using the WHO SORT-IT [19] resources as a guide. These resources were distributed to ToT participants in digital and hard copy once they had completed the training. Microsoft PowerPoint® slide decks were developed for every lecture and plenary session. Speaking notes were included in the “notes” section of each PPT slide to support the delivery of future OR training sessions. The ToT and Facilitators Manual, appendices and slide decks were developed and revised by the UQ Facilitators and the IPHCE PHC programme lead to ensure content was relevant and appropriate to context.

## Training of trainers course evaluation

An electronic pre- and post-course survey was delivered to all participants. Participants were emailed and requested to complete the surveys within 72 hours. The pre-course survey comprised 22 questions (seven multiple-choice and 15 short answer/free text questions) and was designed to gather background information to assist with the delivery of training. The survey included demographic data (name, gender, contact details, education, training experience), preferred training days, experience with online learning, and personal expectations for the forthcoming training.

The post-course survey comprised 12 questions (six Likert response and six short answer/free text questions) and was designed to evaluate participant satisfaction and usefulness of training undertaken by the participants. The survey included demographic data (name, gender), quality, style and delivery of training and how participants will apply knowledge. Neither survey was compulsory.

## Training of trainers course evaluation analysis

Quantitative and qualitative data were extracted from and manipulated using Microsoft Excel® (Version 2202) database and simple descriptive analysis performed. Open-ended questions were compiled into areas of similarity categorised according to themes.

## Results

### Operational research training of trainers participants

Twelve participants were involved in the OR ToT training with six participants from IPHCE and six from universities with formal links to IPHCE. Participants were from diverse backgrounds ranging from programme directors, research advisors, research officers, lecturers and senior academics. Four SORT-IT graduates from Ethiopia agreed to act as mentors to the OR ToT trainers and for future OR courses. Attendance at training varied as some participants were engaged in other concurrent training activities. Training times were flexible to allow maximal numbers of participants to attend training. Facilitators attended all training sessions. One facilitator was responsible for leading participant discussion, while the other monitored the chat function and provided technical assistance. This necessitated several one-on-one interactions using screen share modality to allow observation of the process undertaken by participants.

The OR protocols developed by the participants in their four groups were completed to the draft stage. Ethics approval for each protocol was acquired from the appropriate organisation (IPHCE and affiliated universities). As participants were senior staff, they did not have direct access to required data so also required funding to employ staff to access data at the community (woreda) level. Funding applications were provided to the funding body (AIHA). Ethics submissions were unable to be processed until funding was confirmed.

### Operational research on ‘training of trainers’ trainer resources

Feedback on the content and cultural appropriateness of the ToT and Facilitators Manual (see Table 2) was sought from the IPHCE Primary Health Care programme lead. The IPHCE Programme Lead suggested that some additional clarification regarding participant requirements, such as minimum tertiary qualifications for future OR courses was required. The developed Facilitators Manual had similar requirements to the existing SORT-IT programme excluding the need to provide a written commitment to attend all modules and implement course knowledge for a minimum of 18 months. Compliance with a Code of Conduct in accordance with IPHCE values was expected of all participants.

### Training content and delivery

Training was conducted using face-to-face training in Ethiopia for the first session and the remainder via Zoom videoconferencing. Attempts to use participant webcams to improve group cohesion were abandoned due to poor internet connectivity. Breakout rooms were used to allow participants to undertake group discussions. The facilitators moved between the breakout rooms and engaged and prompted discussion. Participants were encouraged to share the role of providing feedback to the whole group with most participants engaging frequently during each session. The varied roles and experience of participants and mentors allowed them to share knowledge and practical experience.

### Course surveys

The pre-course survey received 5 out of 12 responses (42%), comprising three males and two females. All males had Doctor of Philosophy qualifications and females had a Master’s degree. All respondents had leadership and/or programme coordination roles and expected to improve their knowledge in project management. Three of the respondents highlighted that the IPHCE is responsible for capacity building at community (Woreda) level with two identifying strategies from community/national level to globally. No respondent identified any challenges in their current roles. Respondents were eager to complete some training using the hybrid model, ‘good to deliver some part of the training face-to-face modality or blended for better interaction (Respondent 1)’.

The post-course evaluation survey was emailed to 12 participants. A total of eight (67%) participants (six male and two female) completed the survey. Of those that responded, participants were satisfied (*n* = 5, 63%) or very satisfied (*n* = 2, 25%) with the teaching quality. One participant stated they were strongly dissatisfied with the teaching quality. This response was contradictory to other written comments that stated they liked how the training was offered. All respondents (100%) agreed or strongly agreed that the training met their learning needs and helped their learning. All respondents (100%) agreed or strongly agreed that they were satisfied with the online training format and 75% (*n* = 6) agreed or strongly agreed that the Blackboard online platform helped support their learning. The remaining two respondents neither agreed nor disagreed regarding whether the online platform helped support their learning. All respondents (100%) agreed or strongly agreed that the training will help them in their role. Respondents provided examples of how they would be able to apply their training knowledge, ‘It will help me to train other people in OR, correctly identify problems in programme implementation and recommend ways of implementing them (Respondent 1)’, and ‘ … operational research is a key evaluation mechanism … I will apply it in our settings to convince policy makers and planners (Respondent 2)’, and ‘The training is also very important to directly conduct OR during our support to improve the PHC system of Ethiopia (Respondent 5)’. Participants from one hub are planning an OR course to be initiated in 2023.

## Discussion

This case study explores the delivery and evaluation of a hybrid OR ToT programme on PHC implemented through a partnership programme between the IPHCE and UQ. The OR ToT programme resulted in 12 individuals from the IPHCE and affiliated universities undertaking validated OR training using a hybrid model of training. Modifying the existing OR SORT-IT programme allowed it to be updated and adapted to the Ethiopian context. Participants reported they were satisfied with the hybrid training model and found the training to be beneficial in achieving the goals of their organisations. The diverse experience of the participants and mentors provided a learning opportunity for others in a non-threatening environment. Working with other participants on the protocol fostered professional relationships. The group work throughout the training enabled participants to get to know each other and support each other when conducting training in the future.

### Training delivery and technology

Delivering training online has been more accepted since the SARS CoV2 pandemic with technology identified as challenging [21]. Limiting the use of cameras prohibited facilitators and participants to read facial cues with some social interactions stilted. The use of the chat function was accepted and allowed anonymity if desired. The use of the Blackboard learning platform lead to some difficulties with its use. The facilitators were able to assist all participants with any technology difficulties. In retrospect, providing a dedicated session for enrolment into all platforms at the beginning of the training would have been useful. Additional one-on-one sessions could be provided at this time to ensure all participants have access to all resources.

### Protocol development and data collection

All participants contributed to protocol development and development of data collection tools during planned facilitated sessions. Progress outside of the sessions was limited due to competing work activities. Development of data collection tools was efficient with the diversity of experience of participants, and junior/less experienced staff gaining knowledge and encouragement from more experienced group participants and course mentors. Senior staff not directly working at programme level identified their limitations with data collection at the community/woreda level. Course participants were less suitable for data collection at two levels; firstly, from a seniority level, their roles precluded time away from their substantive position. Secondly, from a community position, their lack of knowledge regarding the process may have missed valuable data due to lack of familiarity with data collection procedures at the community level. The direct involvement of senior course participants at the community level (staff communication and data collection) may have resulted in a social desirability bias. Community staff may have been compelled to respond with more favourable data rather than provide the complete data set. Investigating funding sources at the beginning of training would have facilitated the protocol process and supported participants. The SORT-IT programme is generally completed within 12 months. An accelerated course format was deliberately initiated to ensure that the hands-on mode was experienced by the participants of this ToT course. Reflecting on this process highlighted that once draft ethics submissions were completed, participants began to identify some of the challenges with data collection, such as identifying appropriate stakeholders, and the need for working with health workers at the coalface. Completion of the OR ToT course resulted in participants who were confident in mentoring and had greater insight into some of the challenges that future participants may experience. The pre-and post – course evaluation response rates were limited and may have been influenced by the inclusion of their name. Less than half (42%) completed the pre-course survey, which focused on experience and expectations. The post-course evaluation was completed by 75% of participants and while very positive, this may not be consistent among the non-respondents.

### Mentorship

The diverse experience of participants and inclusion of mentors who had previously completed the SORT-IT programme provided participants with knowledge in developing and refining research skills. Group work activities allowed participants to receive constructive feedback and develop academic writing skills. It is possible that the expedited online course may have limited some participants with their engagement during group work with more senior participants [22]. The use of the more traditional SORT IT programme may have allowed participants to develop stronger relationships, albeit in a longer timeframe. Indeed, the traditional SORT-IT approach utilises the previous course participants as ‘junior’ mentors to work alongside more experienced trainers to gain knowledge of the requirements to implement a course. Our approach was designed to short-cut this process using a ToT approach.

### Logistics

Operational research training was one of the four components of the project being implemented between the IPHCE and UQ. All components involved training and meetings with key personnel. Some participants were involved in training in one or two other components. Participants sometimes had two training sessions scheduled for the same times. Session times were rescheduled to maximise attendance when available.

The SORT-IT repository [23] and training videos [19] provides a wealth of information which is easily available. Providing training using examples specific to the country where it is delivered may have a greater impact on participants.

## Conclusion

This is the first time that an OR ToT course has been delivered in a hybrid (face-to-face and virtual) model. The experience of the participants allowed them to share skills and knowledge while also developing a greater understanding of OR and strategies to enhance future training which will contribute to building capacity within Woredas and the communities. Overall, the ToT course will contribute to improving skills and knowledge of health and research personnel, improve health care delivery and contribute to policy change.

This article provides an alternative for delivering OR training for researchers/primary health care practitioners in low- and middle-income countries. This model promotes collaboration with supporting organisations, such as the WHO and recognises the diversity of skills in the workplace. This model helps to promote collaboration and knowledge sharing to help build capacity where required.

## Paper context

A hybrid ‘Training of Trainers’ approach for the WHO Structured Operational Research Training Initiative (SORT-IT) was facilitated by a partnership between the International Institute for Primary Health Care, Ethiopia and The University of Queensland, Australia. Training was conducted over three months using existing and developedcontext specific resources, including a facilitation manual. Participants completed training understanding requirements and challenges of delivering future courses. A hybrid model was suitable for sharing of skills and knowledge.
